# Development of an open-source web-based intervention for Brazilian smokers – Viva sem Tabaco

**DOI:** 10.1186/s12911-016-0339-7

**Published:** 2016-08-02

**Authors:** H. P. Gomide, H. S. Bernardino, K. Richter, L. F. Martins, T. M. Ronzani

**Affiliations:** 1 Universidade Federal de Juiz de Fora, Juiz de Fora, Brazil; 2 The University of Kansas Medical Center, Kansas City, KS USA

**Keywords:** Smoking cessation, E-health, Internet, Intervention, Health education

## Abstract

**Background:**

Web-based interventions for smoking cessation available in Portuguese do not adhere to evidence-based treatment guidelines. Besides, all existing web-based interventions are built on proprietary platforms that developing countries often cannot afford. We aimed to describe the development of “Viva sem Tabaco”, an open-source web-based intervention.

**Results:**

The development of the intervention included the selection of content from evidence-based guidelines for smoking cessation, the design of the first layout, conduction of 2 focus groups to identify potential features, refinement of the layout based on focus groups and correction of content based on feedback provided by specialists on smoking cessation. At the end, we released the source-code and intervention on the Internet and translated it into Spanish and English.

**Conclusions:**

The intervention developed fills gaps in the information available in Portuguese and the lack of open-source interventions for smoking cessation. The open-source licensing format and its translation system may help researchers from different countries deploying evidence-based interventions for smoking cessation.

## Background

Tobacco use causes approximately 5.4 million deaths a year worldwide [1]. It is estimated that between one-third to one-half of the smokers die due to diseases associated with cigarette smoking and that smokers live an average ten years less than non-smokers [2]. The World Health Organization recommends that a variety of treatments modalities can be offered to smokers [3].

Web-based interventions are a promising tobacco treatment modality [4, 5], especially in developing countries with poor access to health resources [6]. Meta-analyses demonstrate that web-based interventions for smoking cessation are cost-effective [7, 8]. Web-based interventions are usually self-guided and automated [7]. Unlike conventional treatments, these interventions can be used simultaneously by many smokers and provide round-the-clock access. Besides, web-based interventions can help smokers who are on waiting lists for in-person treatment and those who live far from health care centers [6].

The potential benefits of these interventions are increasing in developing countries due to the growth of the Internet access and consumers’ ability to independently search for health information. For example, in December 2013, Brazil had approximately 110 million Internet users [9], of whom approximately 43.0 % sought health-related information [10]. In addition to the growing number of Internet users, there is also a large demand for tobacco treatment in Brazil, as 45.6 % of smokers have made at least one attempt to quit smoking, and less than half of smokers receive advice from health professionals to quit [11].

In general, researchers develop digital interventions using a “black box” model, in which they create and test interventions in controlled trials, with little input from end users or interim evaluation [12]. There are no widely-accepted guidelines for the development of web-based interventions for health interventions, including smoking cessation. Recommendations proposed for development of web-based interventions include (a) soliciting active participation of users [13], (b) complying with usability guidelines [14] and (c) optimizing content for search engines [15]. To evaluate tobacco cessation-specific interventions, researchers have adopted guidelines for face-to-face treatment [16]. Besides, Instructional Design [17] and the Persuasive System Design [18] are proposed as frameworks for developing web-based interventions. The later has been shown to be a useful framework to increase intervention adherence [19].

Despite the great number of web-based interventions for smoking cessation, no one out of 60 evaluated by Bock et al. [16] was licensed under an open-source format. Open-source licenses function equally well in many applications (e.g., Linux, Mozilla Firefox, Wordpress). The use of open-source licenses ensures free access to the software source code and could facilitate replication and adaption by public health practitioners and researchers worldwide.

Information about health on the Internet can be unreliable [16, 20]. Carlini et al. [20] assessed the coverage and the quality of web-based interventions available in Portuguese. Replicating the methodology and guidelines originally developed by Bock et al. [16] to assess web-based interventions for smoking cessations, Carlini et al. [20] found that none of the eleven interventions available in Portuguese adequately addressed all components Bock’s guideline. Moreover, some interventions included potentially harmful information, such as advising smokers to enjoy simply smoking a cigarette in the event of a lapse, suggesting that medications not be helpful in the process of quitting, or recommending that smokers keep smoking if they are unsure about the benefits of quitting. Also, most websites did not meet the basic reliability criteria (e.g., provide clear purpose, declare sources of funding, and refer to content’s source) of the Health on the Net Foundation [21], a Code of Ethics on publishing health information on the Internet.

The current study describes the development of “Viva sem Tabaco”, an open-source Internet-based smoking cessation intervention. “Viva sem Tabaco” sought to address the shortcomings of prior Portuguese-language web interventions by collecting end-user input from the start, enlisting tobacco control experts to evaluate the content, adhering to the Health on the Net Foundation Code of Ethics [21], using content from treatment guidelines and meta-analyses [5, 22–24].

## Implementation

**Fig. 1:**
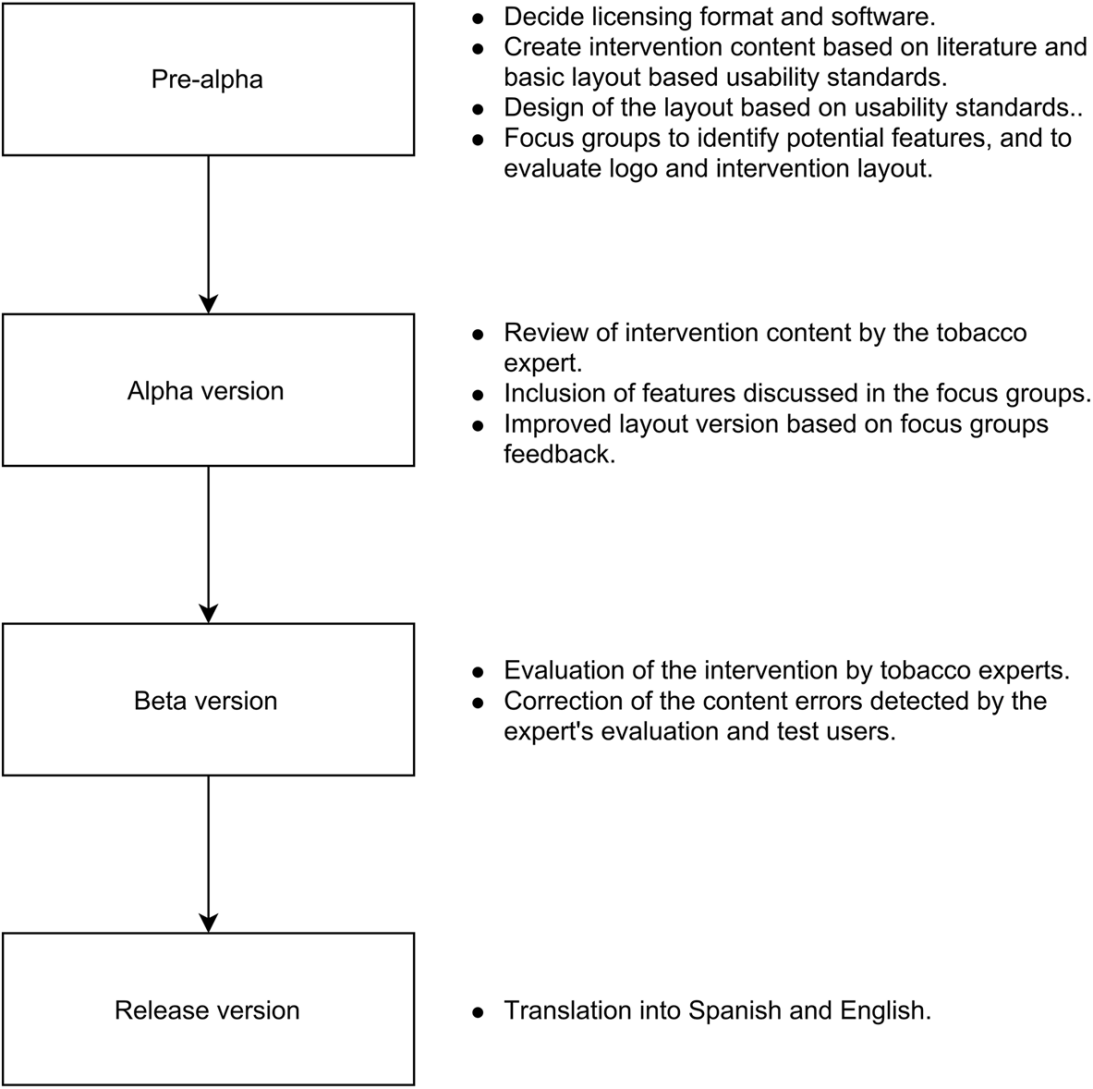
Description of the development phases of "Viva sem Tabaco"

We divided intervention development into four phases, which corresponded to versions in the development process: Pre-alpha, Alpha, Beta and Release Version (Fig. 1). Pre-Alpha included selecting the license format, software, and content; designing the intervention layout; conducting focus groups to select potential features. Alpha included reviewing the content, including features identified on focus groups, and refining the layout. Beta included evaluating the function and content of the web intervention and correcting errors. At the close of development, we released the intervention on the Internet and translated it into Spanish, English and German.

## Pre-Alpha version

### Licensing format and choice of software

Within the first development phase, we decided to use the GNU General Public License because it guarantees four freedoms: 1) to use the software for any purpose, 2) to change the software content to suit anyone’s needs, 3) to share the software with friends and colleagues, and 4) to share the changes make by everyone [25]. We also decided to use “freeware” for all intervention development to enable other researchers and developers to reproduce and adapt the intervention.

All graphs, images, flowcharts, video animation were also licensed under the GNU license and made available on our Github account. We decided to use the Java JSF to write the source-code; MySQL, to store data, and the Glassfish as the application server. The code was created in the NetBeans IDE. To create images, we used the Inkscape and Gimp; to create the video animation, Blender, and Openshot; to create the flowcharts and the initial content, Dia and Libreoffice Writer. The content was written and developed on Debian-Linux based computers.

### Offline draft of the intervention content

After determining the software and the license format, we created the intervention content based on tobacco treatment guidelines [24, 26] and meta-analyses published by the *“Cochrane Tobacco Addiction Group”* [5, 22, 23]. The intervention structure was developed by adapting the “Transtheoretical Model of Behavior Change” [27]. This model divides people with health conditions into phases according to their degree of motivation for change (i.e., pre-contemplation, contemplation, preparation, action, and maintenance). Accordingly, the intervention content was structured into three major content areas: “Is it worth stopping smoking?” (Precontemplation/Contemplation), “Are you ready to quit?” (Preparation/Action), and “Have you already stopped?” (Maintenance).

Content in the “Is it worth stopping smoking?” section was designed to enhance the motivation of smokers and increase the likelihood of a quit attempt. Smokers in the “Ready to quit?” section were prompted to develop a quit plan. The “Have you already stopped?” section focused on helping smokers who had stopped avoid or recover from a slip or relapse.

### Design of the main layout and brand

We also drafted the format of the intervention (e.g., headers, footers, and main style), the brand and name. The overall design was based on general usability guidelines proposed by the U.S. Department of Health Services [24]. We also accommodated characteristics of Brazilian Internet and computer use such as commonly used browsers versions, connection speed and type (ADSL, cable), the level of ability to use computers and top browsing difficulties [10].

### Focus groups

To help guide content and formatting decisions, we conducted two focus groups to understand users’ perceptions of web-based interventions. The study was approved by the Institutional Review Board of the Federal University of Juiz de Fora, n. 033/2011, protocol CEP UFJF 2293.033.2011. After the focus group, smokers received a brief counseling session and referral to a free smoking cessation program. All material (e.g., transcripts, focus groups guide, and the detailed report) related to the focus groups is freely available elsewhere [28].

### Participants

In total, seven participants met inclusion criteria, which were: (a) to be a regular smoker or to be attending tobacco treatment; (b) to be older than 18 years, and (c) to use the Internet from a desktop computer or a notebook for at least 1 h a week. Smokers were recruited via university-based advertisements and social network campaigns(5) and cessation groups in two health facilities(2). Before the focus groups, all participants received a brief description of the research, and signed the written consent form.

### Methods

The focus groups were conducted by two researchers who had had previous experience conducting focus groups (HG, MA in Psychology, and LM, MA in Psychology), using a focus group guide. Questions were open-ended and permitted follow-up prompts to gather information in a semi-structured format. Topics included how participants use the Internet, how they seek information about health and what methods they use to identify reliable information. Other topics of interest were users’ attitudes towards web-based interventions and their opinions regarding interactive components such as forums, tests, diaries, and teleconferences. Participants were asked about the positive and negative features of exemplar websites. Each session lasted approximately one hour and took place at the research center in the university campus. The focus groups audio was transcribed by one of the authors (HG). The authors, HG and LM, then summarized participant comments according to major topic areas covered by the question guide.

### Results

The median age of the participants was 40 years, and the interquartile range was 22.5 years. Six out of the seven participants did not finish their college studies. The median of hours using the computer per week was 40 h.

When participants were asked how they search for health information on the Internet, they noted that search tools were their main strategy. They evaluate the quality of the information they find by looking for how consistent the information is across different websites, evaluating the sources and authors of the information, and asking the opinion of healthcare professionals - “That’s what I generally do, I look for different opinions and check if they make sense” - KM.

In regard to the web-based interventions for smoking cessation, the participants highlighted as relevant tools to be included: tobacco use dependence tests, a how-to-quit guide, and links to social networks “you could create a blog that users can share through social network” - MVS; “or even a website, with a step by step guide” - JAS. The two smokers in cessation treatment focus group stressed the importance of addressing weight gain and tobacco craving. Participants identified the following online tobacco use intervention components as useful: a) information and facts presented in texts and videos; b) tests to evaluate nicotine dependence; c) tools, such as a calculator to calculate expenses with cigarettes. “I’ve made the calculation. It’s been thirty-five days without smoking and I already changed my cellphone” - MVS; and d) social support through social networks. The following components were seen as less useful: a) text messages (SMS), due to the excess of text advertisement and marketing sent to users by the telecoms in Brazil, “If I receive text from some weird number, I already know someone is sending me Spam”- SBO; b) printed diaries, due to the need for daily access and storage, and c) statements from former smokers, which ofter come across as fake.

Participants felt that the content should be developed by healthcare professionals and smokers. The participants liked the program’s proposed name but noted a number of problems with the first layout version. They categorized version one as “cold” and “invasive” because it gave little information about tobacco and asked directly for personal data. They suggested the program be brief because it is difficult for some people to access a computer many times per day. They suggested that intensive interventions could be better implemented via mobile applications.

## Alpha version

At this point, the program consisted of the main design components. The intervention content was written but not translated into code. After the end of the Pre-Alpha phase, we worked with a tobacco treatment expert to refine the intervention components. Based on the expert’s feedback, we made modifications to the intervention flow and content. The main modification involved allowing end users to navigate the intervention content freely, instead of forcing them to follow the intervention’s flow.

We also adjusted the layout to incorporate focus group feedback and potentially improve adherence to the intervention. We included features that would potentially improve adherence to the intervention according to the Persuasive Systems Design Framework [18, 19]. We took into account the three principles of the Framework: primary task support, dialogue support, and social support. Some features that illustrate how we tried to cover the primary task support were a quit plan divided into short pages (tunneling), a calculator to assist smokers to estimate their savings (simulation), and tailored content for users with different levels of motivation (tailoring). Features that illustrate the dialogue support principle were reminders sent through 15 email (reminders), tips on which methods work best for quitting (suggestion), and content written in a way to praise users for their trying to quit effort. The only feature related to social support principle was a page where participants could share their successful stories (Recognition). Also, an introductory video was placed on the main page, and content about weight gain was included. The order of the pages was also changed to enable users to develop a quit plan in a single visit to the website.

## Beta version

### Results

To arrive at the Beta version, we adjusted the Alpha version to incorporate expert and additional focus group feedback. At this point, the program consisted of static and interactive pages, offering a step-to-step quit plan or recover from relapse plan, and email messages.

### Evaluation of Beta version

We invited two specialists to evaluate the content and delivery of the intervention. They were chosen based on their expertise in tobacco treatment, background in research, and because they had no ties to the intervention development project. We used methods developed by Carlini et. al [20]. to structure the assessment. Both experts were instructed on how to complete the “Content Evaluation Questionnaire” and, subsequently, they performed an independent evaluation of the intervention project. We compared their evaluations on an item by item basis; where they conflicted, we enlisted the help of an independent researcher to interpret the discrepancies in numerical and written feedback and provide a score that encompassed both experts’ evaluations.

The Content Evaluation Questionnaire was adapted from Bock et.al [16]. It contained two instruments: *Smoking Treatment Scale-Content* and *Smoking Treatment Scale-Rating.* The first is a list of twelve components of the U.S. Department of Health Services tobacco treatment guideline [24]: (1 and 2) recommend smokers to quit in a strong and personalized manner; (3) assess the readiness to change; (4 and 5) support the quit attempt through a quit plan; (6) offer support throughout the treatment; (7) recommend medications whenever appropriate; (8) provide a face-to-face, by phone or email follow-up, and; (9 – 12) motivate smokers on four areas: the importance of quitting, risks of smoking, rewards of quitting and how to overcome common roadblocks. The second instrument, *Smoking Treatment Scale-Rating,* evaluates web interventions coverage, interactivity, and accuracy according to twelve domains. The first two domains use an ordinal response ranging from 1 (*none*) to 5 (*extensive*). Accuracy is evaluated via an ordinal response of 1–3 (1 = incorrect or potentially harmful, 2 = mostly correct, 3 = totally correct). The specialists also indicated whether each component was interactive or not.

## Results

The evaluation of the experts (Table 1) found the coverage of 9 out of the 12 topics (75 %) to be adequate and three topics provided minimum coverage. Regarding accuracy, seven components were scored as totally correct, and 5 were considered mostly correct. The component with the worst score was the “Offering practical counseling”. The intervention’s interactivity was considered low, two topics out of the 12 scored as interactive.

**Table 1:** Expert’s Evaluation of the "Viva sem Tabaco" intervention by component

Component	Coverage	Accuracy	Interactivity
1. Advise to quit smoking - emphatically	Adequate	Correct	No
2. Advise to quit smoking - personalized	Adequate	Correct	No
3. Assess the readiness to change	Adequate	Correct	No
4. Assess the readiness to quit	Adequate	Correct	Yes
5. Offering practical counseling	Minimum	Mostly correct	No
6. Offering support during treatment	Adequate	Mostly correct	No
7. Recommend pharmacotherapy whenever appropriate	Minimum	Correct	No
8. Face-to-face follow up	Minimum	Correct	No
9. Motivate: importance of quitting	Adequate	Mostly correct	No
10. Motivate: risks	Adequate	Mostly correct	No
11. Motivate: rewards	Adequate	Mostly correct	No
12. Motivate – remove barriers	Adequate	Correct	Yes

## Release version

**Fig. 2:**
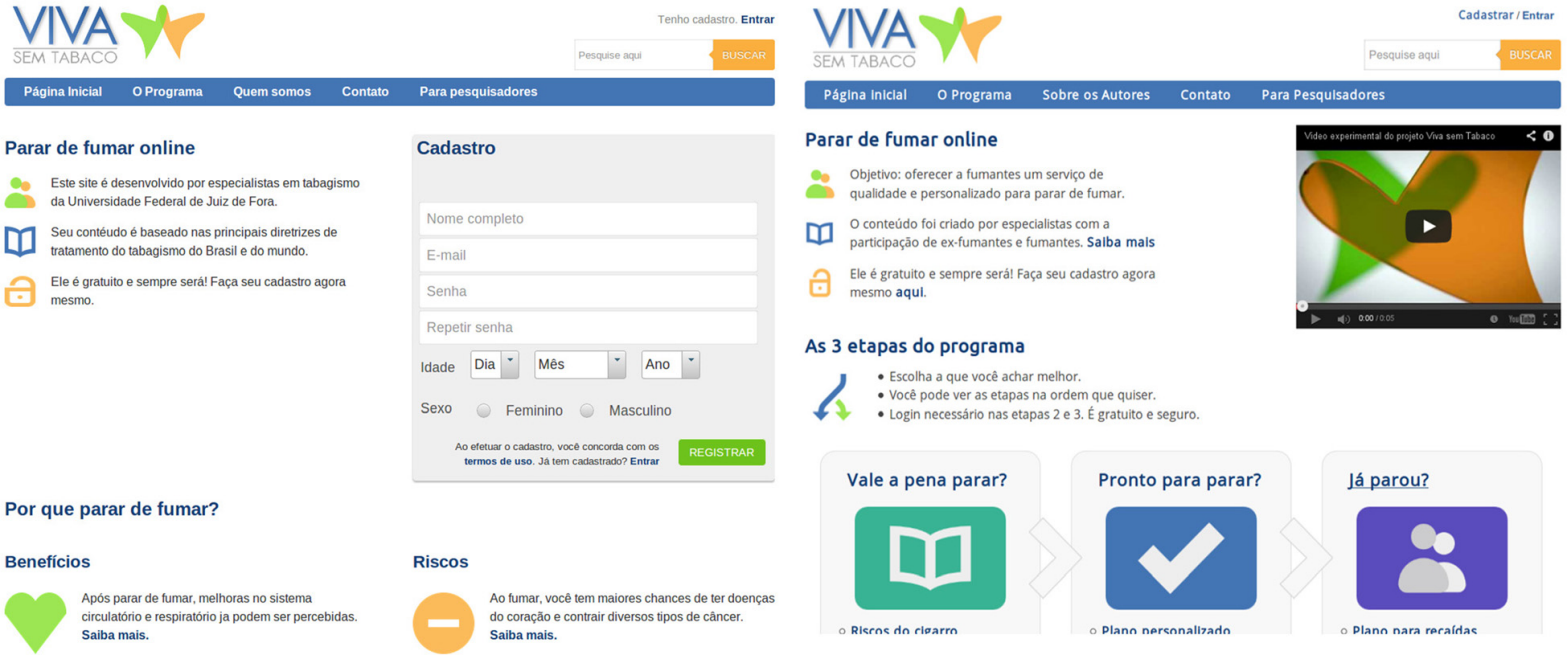
Comparison of the main intervention page between Pre-Alpha Version (left) and Release Version (right)

The intervention’s content was corrected to enhance the strengths and address the weaknesses identified by the experts. Based on the expert feedback, we made improvements like adding questionnaires with feedback to assess the nicotine dependence level and depressive symptoms, increasing the number of follow-up emails and providing further information about the evidence behind the most common quit methods. The development team then reviewed website for compliance with the Health on the Net Foundation principles [21]. We also corrected interface errors identified during informal test runs performed by colleagues. A final list of topics addressed in "Viva sem Tabaco" is displayed on Table 2 and a picture of the interface is depicted on Fig. 2.

**Table 2:** Description of the content of "Viva sem Tabaco" by sessions

Is it worth stopping smoking?	Are you ready to quit?	Have you already stopped?
Benefits of stopping smoking	How to deal with withdrawal	Slips and Relapses
Risks of smoking	Overcoming cravings	Most common relapses causes
Financial costs with a calculator	Medication	Factors that lead to relapse
Withdrawal and craving	Ways to quit	Plan to overcome relapses
Weight gain	Set a quit date	Where to find help
Anxiety and depression	How to avoid relapses	
Doubts about stopping smoking	Weight gain	
Where to find help	Learning from previous quit attempts	
	Quit plan

After the corrections, we adapted the system for internationalization. The content was translated into Spanish by a native speaker/psychologist; English, by a native speaker/expert on smoking cessation; and German, by a native speaker/fluent in Portuguese. The intervention was made available for access at www.vivasemtabaco.com.br, www.vivasintabaco.org, www.livewithouttobacco.org, www.lebeohnetabak.org. We published the source code of the intervention at http://github.com/crepeia/wati.

### Behavior change techniques

In the final version, we have applied different behavior change techniques according to the BCT Taxonomy [29]. For the session “Is it worth stopping smoking?” we added: information about health consequences (5.1), instruction on how to perform the behavior of quitting (4.1), feedback on behavior (2.1) when filling in anxiety and depression questionnaires, (10.9) self-reward. For the session “Are you ready to quit”, information about health (5.1), emotional consequences (5.6), focus on past success (15.3), avoidance/reducing exposure to cues (12.3), restructuring the social environment (12.2), pharmacological support (11.1), reduce negative emotions (11.2), comparative imagining of future outcomes (9.3), remove access to the reward (7.4), action planning (1.4), goal setting (1.3), problem solving (1.2). For the “Have you already stopped” session, problem solving (1.2), goal setting (1.3), action planning (1.4), and focus on past success (15.3).

## Discussion

"Viva sem Tabaco" fills an important gap in evidence-based interventions for smoking cessation available in Portuguese and also a gap in open-source web interventions. It offers Brazilian smokers a self-help method to quit smoking that complements the methods currently available in the country. To promote the intervention’s adoption in other countries, we released almost all related material (images, video) under a GPL license. Its open-source format facilitates its translation and adaptation to other languages without the need of developing a new system. We are, at present, mining user data to improve navigation flow, building a recommendation system and conducting a randomized trial to examine its effectiveness in helping tobacco users quit.

From a public health perspective, "Viva sem Tabaco" and other web interventions might be important resources for patients on waiting lists for clinic-based care or could be offered as a complement to traditional cessation treatment. Such websites could also reduce barriers to treatment raised by physical disabilities, low income, or stigma. More will have access to web interventions as the number of Internet users grows. Thus, these interventions can potentially enhance current health promotion and prevention activities [6].

Compared to the interventions available in Portuguese evaluated by Carlini et.al [20], “Viva sem Tabaco” provides better topic coverage and adhered better to the code of conduct of the Health on the Net Foundation [21]. The use of the Transtheoretical Model [27] for behavior change facilitated the division of the content to meet smokers at their stage in the cessation process.

The development of "Viva sem Tabaco" can serve as a simplified guide for how researchers and health advocates can develop other web-based health interventions. The four-phase process facilitated the development of an accessible intervention based on scientific evidence. The participation of end users and specialists during development phases strengthened the organization and content of the program.

The process reported here has some limitations. First, we only held two focus groups, and participants may have not been representative of the end-users. Smokers who seek face-to-face treatment may differ from those who use web-based interventions [10, 11]. The small numbers of participants resulted from the difficulty of finding smokers who met the inclusion criteria and who were able to get to the research center. User navigation behavior data could provide additional insight into this issue. Second, the resulting website might not be effective or useful for all smokers. In Brazil, Internet users have higher education than the general population [30]. Third, the evaluation of the content was based on the guidelines for clinical practice that were created for face-to-face and telephone treatment. Fourth, the content evaluation was performed by specialists. Evaluation by the lay public with different levels of reading could provide other important insights regarding content and format. Fifth, the Transtheoretical Model as a guide for the quitting process has come under criticism [31]. However, the literature points out that the interventions for tobacco use based on stages present the same effectiveness of those based on “readiness” stages [22] and little is known about which theoretical references and components are more effective in the web-based intervention modality for smoking cessation [5, 32]. The use of design frameworks such as the Persuasive System Design [18] and a common taxonomy for behavior change techniques [29] during the development of web-based interventions for smoking cessation are good targets for further studies.

Although the focus groups provided insights that helped improve the intervention layout and content, further qualitative and quantitative studies should solicit data on the user experience from the actual end user of the intervention. Our current trial of “Viva sem Tabaco” will evaluate its effectiveness in prompting quit attempts and helping tobacco users achieve abstinence. Besides assessing the intervention’s effectiveness, further studies may help advance the science by assessing the utility of specific features such as interactivity and the use of additional components like weight gain reduction, and relapse prevention techniques. Future studies on project development could employ techniques such as Data Mining and Machine Learning, which may lead to better understanding of how smokers use websites.

## Conclusions

The present study describes the development process of the first open source web-based intervention for smoking cessation that is compliant with the current guidelines for face-to-face treatment. The open-source infrastructure should help researchers develop web-based interventions for smokers in other languages or via other platforms, such as mobile applications. The intervention fills gaps in the information available in Portuguese on the Internet and the lack of open-source interventions for smoking cessation.

## Availability and requirements

- **Project name:** Viva sem Tabaco: an open-source web-based intervention for smoking cessation
- **Project home page:**https://github.com/crepeia/wati/
- **Operating System(s):** Platform independent
- **Programming language:** Java, JSF
- **Other requirements:** Java 1.6. or higher, Glassfish 3.x
- **License:** GNU GPL1.3
- **Any restrictions to use by non-academics:** No

## Abbreviations

GPL, General public licence
